# Metastatic Neuroblastoma Presenting as Suspected Septic Arthritis: A Case Report

**DOI:** 10.7759/cureus.117640

**Published:** 2026-10-08

**Authors:** Rayan Aljubeh, Christeen Sourial

**Affiliations:** 1 College of Medicine, University of Sharjah, Sharjah, ARE; 2 Division of Pediatric Hematology and Oncology, Department of Pediatrics, Sheikh Khalifa Medical City, Abu Dhabi, ARE

**Keywords:** diagnostic challenge, limping child, musculoskeletal symptoms, neuroblastoma, pediatric oncology

## Abstract

Neuroblastoma is the most common extracranial solid malignancy in childhood and can present with a wide range of clinical manifestations. Although metastatic disease frequently involves the bone and bone marrow, musculoskeletal symptoms may mimic common orthopedic conditions, resulting in diagnostic delays. We report a case of a previously healthy two-year-old boy who presented with a three-week history of bilateral lower limb pain, limping, intermittent fever, and abdominal pain. Initial evaluation raised suspicion for septic arthritis due to elevated inflammatory markers and magnetic resonance imaging (MRI) findings suggestive of hip infection. Hip aspiration yielded turbid inflammatory fluid containing numerous pus cells. However, cultures remained sterile, and the patient showed persistent symptoms despite broad-spectrum antimicrobial therapy. Further evaluation with whole-body MRI revealed diffuse bone marrow signal abnormalities involving the axial and appendicular skeleton, together with a right suprarenal mass, findings highly suspicious for metastatic neuroblastoma. Subsequent positron emission tomography/computed tomography performed at an external institution demonstrated widespread skeletal involvement, further supporting the suspicion of metastatic neuroblastoma. Tissue diagnosis was not obtained at our institution because the family elected to pursue further evaluation and treatment abroad. This case highlights the importance of reconsidering the diagnosis in children with presumed musculoskeletal infection who fail to improve as expected. Persistent fever, sterile cultures, anemia, elevated inflammatory markers, and multifocal musculoskeletal symptoms should prompt consideration of malignancy and early multidisciplinary evaluation.

## Introduction

Neuroblastoma is the most common extracranial solid malignancy in childhood and accounts for approximately 8-10% of all pediatric cancers. It originates from primitive neural crest cells and most commonly arises in the adrenal medulla or sympathetic chain. The disease demonstrates a highly heterogeneous clinical course, ranging from localized tumors with excellent prognosis to widely metastatic disease with poor outcomes, depending on age at diagnosis, biological characteristics, and stage at presentation [1,2]. Approximately half of patients present with metastatic disease at diagnosis, with bone and bone marrow being the most frequent sites of dissemination. Consequently, skeletal involvement may dominate the clinical presentation, leading to symptoms such as bone pain, limping, refusal to bear weight, irritability, or generalized musculoskeletal discomfort [3]. These manifestations are particularly challenging in young children, in whom symptoms are often nonspecific and overlap with far more common orthopedic and infectious conditions. Musculoskeletal presentations of neuroblastoma can closely mimic septic arthritis, osteomyelitis, or transient synovitis, often leading to initial misdiagnosis and treatment with antibiotics or even surgical intervention. Several case reports and small case series have highlighted children who were initially managed for presumed septic arthritis of the hip before the correct diagnosis of metastatic neuroblastoma was established [4,5]. Because these atypical musculoskeletal presentations closely mimic far more common orthopedic and infectious conditions, delayed diagnosis is not uncommon. In such cases, persistently negative microbiological cultures, lack of sustained clinical response to antibiotics, and the presence of systemic features such as anemia or elevated inflammatory markers may provide important diagnostic clues. Early recognition of these atypical presentations is therefore critical, as diagnostic delays may lead to disease progression and adversely affect outcomes. Advanced imaging modalities such as magnetic resonance imaging (MRI), whole-body MRI, and positron emission tomography/computed tomography (PET/CT) play an essential role in identifying multifocal bone involvement and occult primary tumors in cases where clinical findings are misleading [3]. We report a case of a previously healthy two-year-old boy who presented with fever, limping, and lower limb pain initially suggestive of septic arthritis but was ultimately found to have imaging and laboratory findings highly suspicious for metastatic neuroblastoma with diffuse skeletal involvement. Although several reports have described neuroblastoma presenting as presumed musculoskeletal infection, such presentations remain uncommon, and additional well-documented cases are valuable for increasing awareness of the clinical features that may facilitate earlier recognition.

## Case presentation

A previously healthy two-year-old boy presented with a three-week history of bilateral lower limb pain associated with limping, intermittent high-grade fever (up to 39°C), abdominal pain, decreased activity, and reduced appetite. The illness initially began with fever and upper respiratory tract symptoms, for which he received oral antibiotics at an outside healthcare facility. Despite treatment, his fever persisted, and he developed progressive difficulty with weight-bearing.

The patient underwent evaluation at multiple healthcare facilities. Initial plain radiographs of the pelvis and bilateral lower extremities were unremarkable. Laboratory investigations demonstrated elevated inflammatory markers, with C-reactive protein (CRP) increasing from 11 mg/L to 116 mg/L before decreasing to 83.8 mg/L, and erythrocyte sedimentation rate (ESR) increasing from 37 mm/hour to 105 mm/hour. Procalcitonin was negative, and blood cultures remained sterile. Ultrasonography of both hips showed no evidence of joint effusion.

MRI of the right hip demonstrated a moderate joint effusion with marrow edema involving the right iliac bone and surrounding inflammatory changes, raising suspicion for septic arthritis, while transient synovitis was considered less likely. The patient was admitted to an outside tertiary hospital, where right hip aspiration yielded turbid inflammatory fluid containing numerous pus cells, raising concern for septic arthritis. However, microbiological cultures remained sterile despite the inflammatory findings. He was treated empirically with intravenous vancomycin and ceftriaxone for seven days, followed by oral amoxicillin-clavulanate and rifampicin.

Despite antimicrobial therapy, the patient continued to experience intermittent fever and persistent abdominal pain. Repeat MRI demonstrated edema and enhancement involving the myotendinous junction of the right rectus femoris, intermuscular edema along the iliopsoas muscle, and minimal right hip joint effusion with synovial enhancement. No evidence of osteomyelitis was identified, and repeat ultrasonography of both hips remained normal. Owing to persistent symptoms despite treatment, he was referred to our institution for further evaluation.

On presentation to our institution, the patient appeared alert and mildly distressed. He was afebrile (37.2°C), with a heart rate of 123 beats/minute, respiratory rate of 23 breaths/minute, blood pressure of 96/56 mmHg, and oxygen saturation of 99% on room air. Physical examination demonstrated generalized tenderness of both lower extremities with resistance to passive range of motion and refusal to bear weight. Mild abdominal distension was present without tenderness or palpable masses. The remainder of the examination was unremarkable.

Laboratory investigations demonstrated normocytic anemia (hemoglobin 8.8 g/dL) with markedly elevated inflammatory markers (CRP 174 mg/L), while the white blood cell count remained within the normal range. The initial laboratory results are summarized in Table 1.

**Table 1:** Lab investigations.

Parameter (unit)	Result	Normal Reference Range
Hemoglobin (g/dL)	8.8	11.0-14.0
White blood cell count (×10⁹/L)	8.8	6.0-17.0
Platelets (×10⁹/L)	289	140-400
C-reactive protein (mg/L)	174	0.0-5.0
Sodium (mmol/L)	137	136-145
Potassium (mmol/L)	4.3	3.2-5.5
Urea (mmol/L)	2.4	2.8-8.1
Creatinine (μmol/L)	20	21-42

Abdominal and pelvic radiographs demonstrated fecal loading without evidence of bowel obstruction or acute osseous abnormalities. The patient's abdominal and pelvic radiographs are shown (Figures 1, 2).

**Figure 1:**
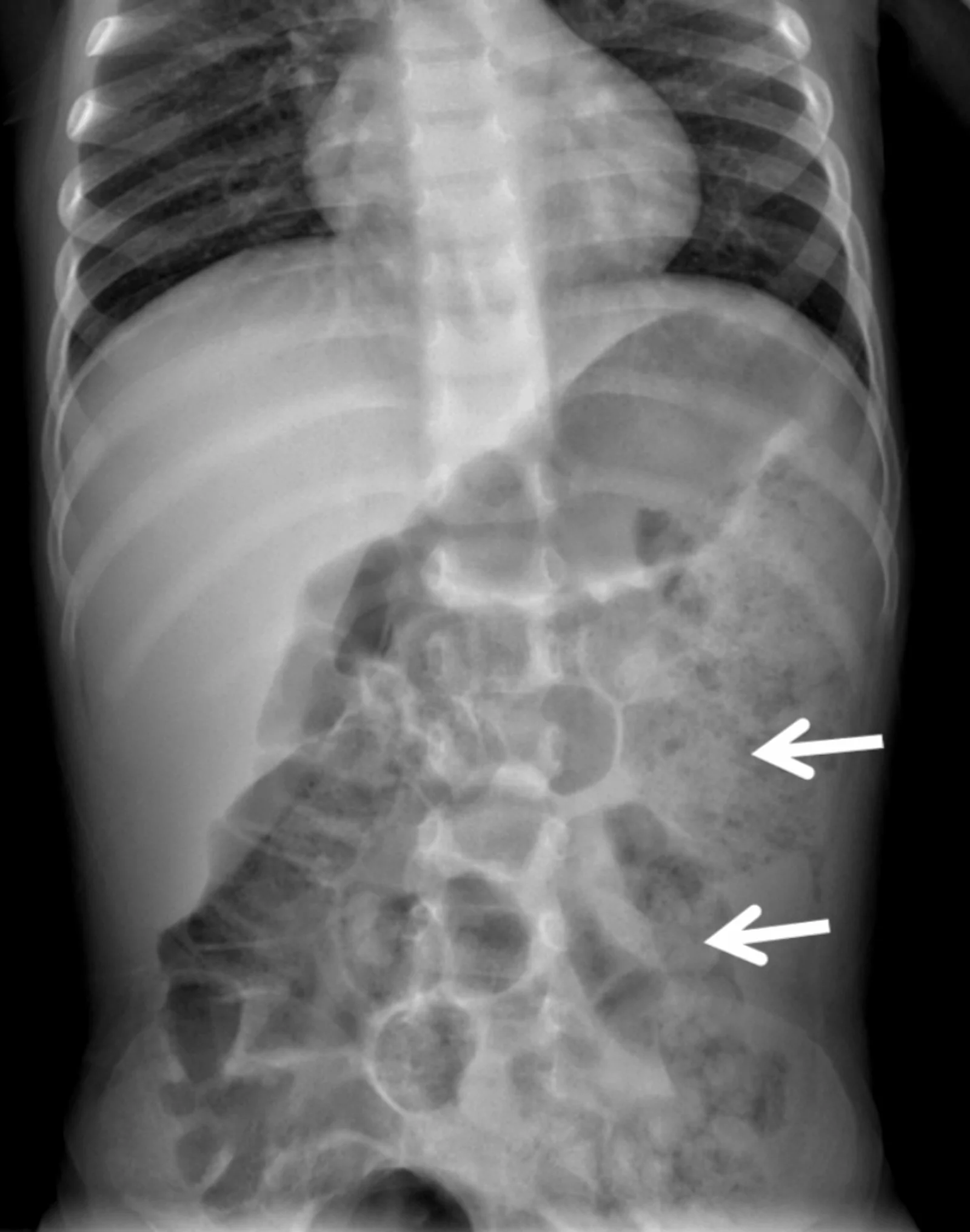
Abdominal radiograph demonstrating fecal loading (arrows) of the colon and rectum without evidence of intestinal obstruction or pneumoperitoneum. Tiny calcific densities are seen in the right upper abdomen medially.

**Figure 2:**
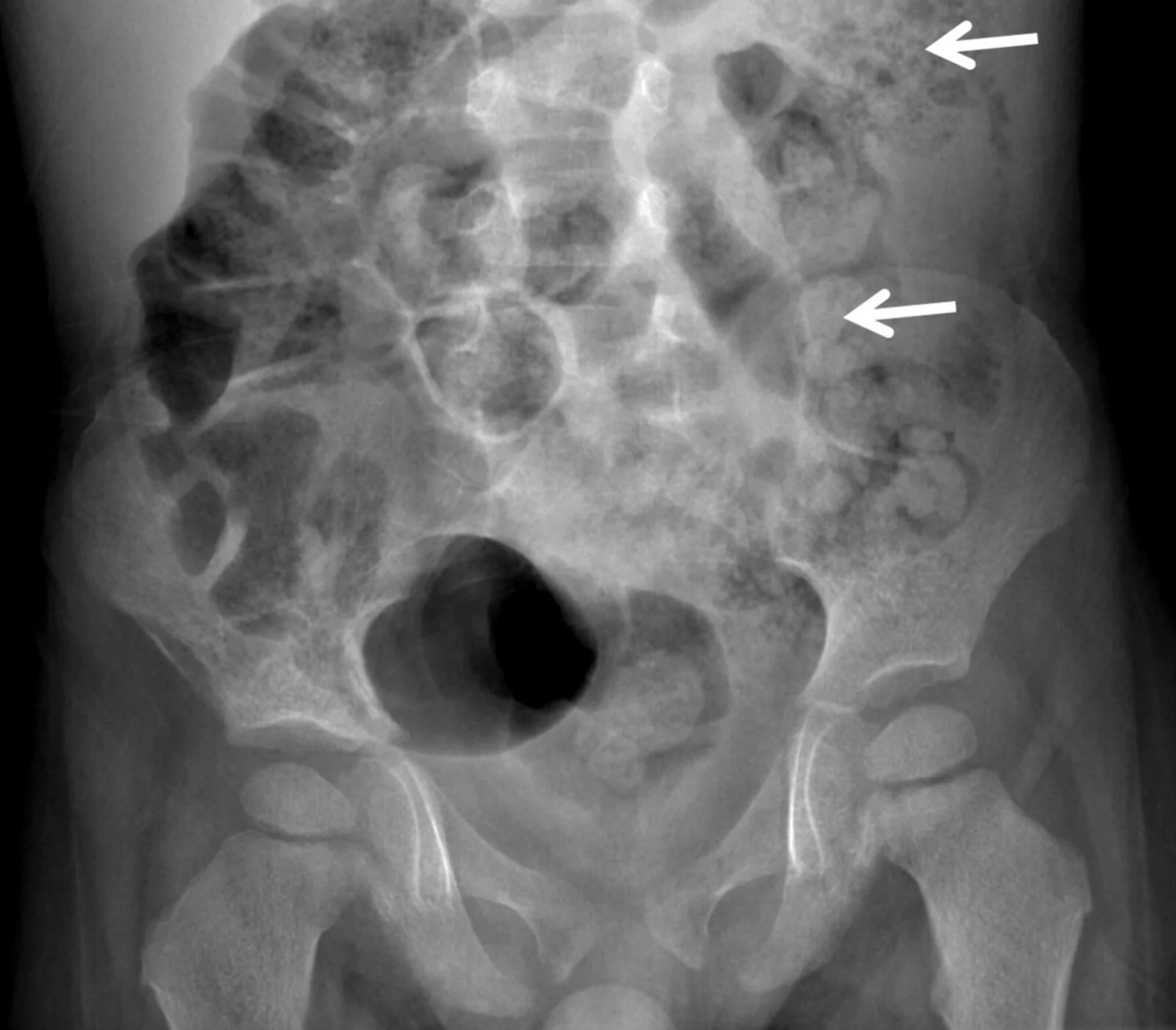
Pelvic radiograph demonstrating normal bony alignment without evidence of fracture, dislocation, or other acute osseous abnormality. Joint spaces are preserved. Fecal loading (arrows) of the colon is noted.

The patient received intravenous fluids, paracetamol, and a fleet enema. Orthopedic review considered septic arthritis unlikely based on the current clinical findings but recommended further investigation. Given the persistent symptoms, markedly elevated inflammatory markers, and marrow edema involving the right iliac bone identified on prior hip MRI, whole-body MRI was performed. This demonstrated diffuse heterogeneous bone marrow signal abnormalities involving the bilateral upper and lower extremities, vertebral bodies, and pelvis, with hyperintense short tau inversion recovery (STIR) signal and hypointense T1-weighted marrow signal. Associated edema was present within the left gluteal and iliacus musculature without a drainable collection. In addition, a well-defined right suprarenal mass measuring 2.7 × 1.4 × 3.7 cm was identified. Whole-body MRI images demonstrating a right suprarenal mass with diffuse marrow signal abnormalities are shown in Figure 3. The constellation of findings was highly concerning for neuroblastoma with diffuse skeletal involvement, while lymphoma remained a less likely differential diagnosis. These imaging findings shifted the clinical suspicion from a primary musculoskeletal infectious process to a disseminated malignant condition.

**Figure 3:**
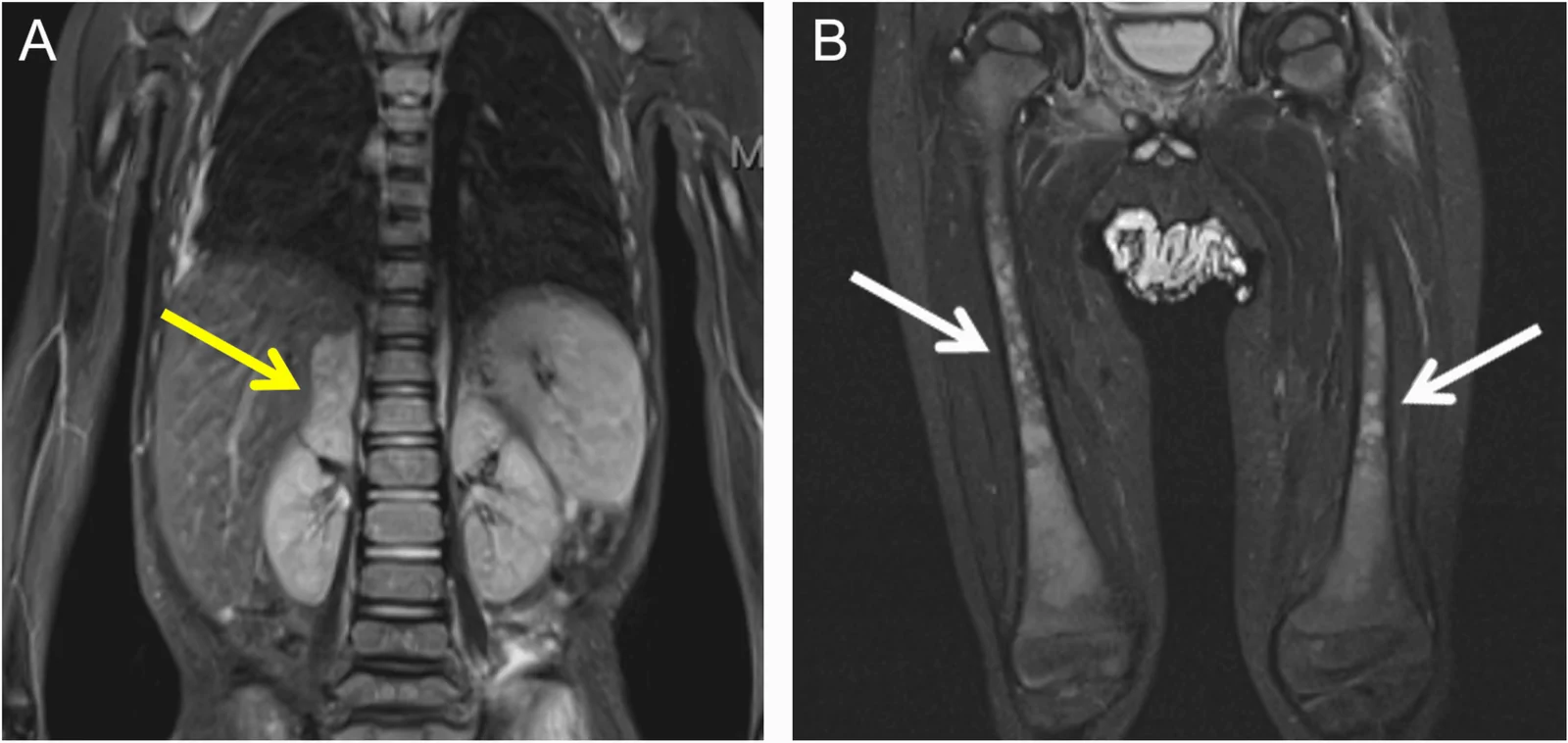
Coronal whole-body short tau inversion recovery (STIR) magnetic resonance imaging (MRI) images. Diffuse heterogeneous bone marrow signal abnormalities are present involving the bilateral upper and lower extremities, vertebral bodies, and pelvis. Bone marrow signal abnormalities in the lower limbs are indicated by white arrows. A well-defined right suprarenal mass measuring approximately 2.7 × 1.4 × 3.7 cm is indicated by the yellow arrow. Associated edema is present within the left gluteal and iliacus musculature without a drainable collection. These findings were radiologically highly suspicious for metastatic neuroblastoma with skeletal involvement.

Pediatric hematology-oncology was consulted, and further investigations demonstrated elevated lactate dehydrogenase (791 U/L), ferritin (1,399 mcg/L), persistently elevated CRP (174 mg/L, increasing to 284 mg/L), normal alpha-fetoprotein and β-human chorionic gonadotropin levels, and elevated urinary vanillylmandelic acid-to-creatinine and homovanillic acid-to-creatinine ratios. The results of the oncologic workup are summarized in Table 2. The findings were considered highly suggestive of metastatic neuroblastoma, and the family was counseled regarding the need for tissue diagnosis through biopsy and subsequent staging investigations.

**Table 2:** Laboratory findings supporting the evaluation for suspected metastatic neuroblastoma.

Parameter (unit)	Result	Normal Reference Range
Lactate dehydrogenase (U/L)	791	135-225
Ferritin (mcg/L)	1,399	10-118
C-reactive protein (mg/L)	284	0.0-5.0
Alpha-fetoprotein (IU/mL)	<2	0-85
β-human chorionic gonadotropin (IU/L)	0.2	<3
Uric acid (µmol/L)	130	212-417
Urinary vanillylmandelic acid-to-creatinine ratio (mmol/mol)	27	<10.3
Urinary homovanillic acid-to-creatinine ratio (mmol/mol)	27	<14.3

The family elected to seek further evaluation and treatment abroad before tissue diagnosis could be performed. At the receiving institution, MRI and PET/CT demonstrated a hypermetabolic right adrenal mass measuring 2.7 × 1.5 × 3.6 cm with widespread lytic skeletal lesions involving the pelvis, femur, tibia, ribs, and ischium, radiologically consistent with metastatic disease. The clinical impression strongly favored metastatic neuroblastoma with diffuse skeletal involvement, although histopathological confirmation had not been obtained. The patient subsequently returned to our institution for continued evaluation. He continued to have intermittent fever, bilateral lower limb pain, and abdominal distension. Repeat laboratory investigations demonstrated persistent systemic inflammation, including elevated CRP (254 mg/L), ferritin (2,252 mcg/L), and abnormal coagulation parameters. Pediatric oncology maintained a high clinical suspicion for metastatic neuroblastoma and recommended biopsy for histopathological confirmation together with completion of staging investigations. Following further counseling regarding the diagnosis and management options, the family again elected to pursue definitive treatment abroad before biopsy could be performed.

## Discussion

Metastatic neuroblastoma frequently involves the bone and bone marrow, and skeletal manifestations may dominate the initial clinical presentation, resulting in symptoms such as bone pain, limping, refusal to bear weight, or joint pain that may obscure the underlying diagnosis [3]. In young children, these nonspecific symptoms may closely resemble more common orthopedic or infectious conditions, creating significant diagnostic challenges.

The present case illustrates how suspected metastatic neuroblastoma may initially mimic septic arthritis and lead to diagnostic uncertainty despite appropriate management of a presumed infection. Our patient initially presented with prolonged fever, progressive lower limb pain, limping, markedly elevated inflammatory markers, and MRI findings concerning for septic arthritis. Hip aspiration yielded turbid inflammatory fluid containing numerous pus cells, further reinforcing an infectious diagnosis despite sterile cultures. Although septic arthritis remains a medical emergency requiring prompt recognition and treatment, persistent symptoms despite appropriate antimicrobial therapy should prompt clinicians to reconsider the diagnosis and broaden the differential diagnosis to include malignant, inflammatory, and other noninfectious conditions.

Several clinical features in our patient served as important clues that the disease course was atypical for uncomplicated septic arthritis. These included persistent fever despite prolonged broad-spectrum antibiotics, repeatedly sterile microbiological cultures, progressive anemia, persistently elevated inflammatory markers, elevated lactate dehydrogenase and ferritin levels, diffuse bilateral musculoskeletal pain rather than isolated monoarticular disease, and ongoing clinical deterioration despite treatment. Although inflammatory markers such as CRP and ESR are commonly associated with infection, they may also be markedly elevated in disseminated malignancy because of tumor-associated systemic inflammation. Therefore, elevated inflammatory markers should not be interpreted in isolation but rather in conjunction with the patient's overall clinical response.

Whole-body MRI proved to be the pivotal investigation in our patient. Rather than confirming isolated joint infection, it demonstrated diffuse bone marrow signal abnormalities involving both the appendicular and axial skeleton together with a right suprarenal mass, findings highly suspicious for metastatic neuroblastoma. Subsequent PET/CT performed abroad further demonstrated widespread skeletal involvement, reinforcing the suspected diagnosis. This case illustrates the important role of advanced imaging in children whose clinical course deviates from that expected for musculoskeletal infection, particularly when symptoms persist despite appropriate therapy. Whole-body MRI is particularly valuable in selected patients because it enables comprehensive assessment of the entire skeleton and soft tissues, facilitating the detection of multifocal bone marrow abnormalities and occult primary tumors that may not be apparent on localized imaging, thereby helping redirect the diagnostic evaluation toward an underlying malignant process.

Our findings are consistent with several previously published reports describing neuroblastoma masquerading as septic arthritis or other orthopedic infections. Lynch and Tomboc reported a three-year-old boy presenting with fever, hip pain, refusal to bear weight, elevated inflammatory markers, and MRI findings initially interpreted as favoring musculoskeletal infection. Despite surgical washout and broad-spectrum antibiotics, persistent symptoms, culture-negative joint specimens, and anemia prompted further evaluation, ultimately revealing metastatic adrenal neuroblastoma with bone marrow involvement [4]. Similarly, Nishiwaki et al. and Alves da Silva et al. described children whose initial presentations closely resembled septic arthritis or an irritable hip, with persistent or atypical clinical features prompting further evaluation that ultimately led to the diagnosis of neuroblastoma [5,6]. More recently, Karray et al. reported a six-year-old boy presenting with isolated lameness whose diagnosis was established after MRI demonstrated extensive bone metastases and abdominal imaging identified an adrenal mass [7]. Collectively, these reports demonstrate recurring warning signs, including persistent fever despite antibiotics, refusal to bear weight, elevated inflammatory markers, sterile microbiological cultures, progressive anemia, persistent limp or gait disturbance, and multifocal bone pain, all of which were also present in our patient.

Beyond individual case reports, our findings are supported by a recent retrospective orthopedic case series evaluating children with biopsy-confirmed neuroblastoma who initially presented with musculoskeletal complaints [8]. In that study, approximately one-fifth of patients first presented to orthopedic services rather than oncology. Persistent hip pain and limping were among the most common presenting symptoms, with several children initially diagnosed with osteomyelitis before biopsy established the diagnosis of neuroblastoma. Most patients demonstrated severe anemia together with elevated ESR, CRP, and lactate dehydrogenase levels, findings that closely parallel those observed in our patient. The authors concluded that persistent or atypical musculoskeletal symptoms, particularly when accompanied by anemia and elevated inflammatory markers, should prompt consideration of neuroblastoma and early tissue diagnosis to reduce delays in treatment. These observations reinforce the importance of maintaining a high index of suspicion for malignancy in children who fail to improve as expected following treatment for presumed orthopedic infection. This conclusion is further supported by a recent systematic review and meta-analysis, which demonstrated that musculoskeletal manifestations are common in childhood malignancies and may delay diagnosis because they mimic orthopedic or rheumatologic disorders [9].

The principal limitation of this report is the absence of histopathological confirmation of neuroblastoma at our institution, as the family elected to pursue further evaluation and treatment abroad before tissue diagnosis could be performed. Although histopathological confirmation remains essential for definitive diagnosis and appropriate risk stratification, the suspected diagnosis in this case was strongly supported by the combination of findings, including whole-body MRI demonstrating a right suprarenal mass with diffuse bone marrow signal abnormalities, elevated urinary catecholamine metabolites, PET/CT findings demonstrating widespread skeletal involvement, and multidisciplinary pediatric oncology assessment. The absence of tissue confirmation represents an important limitation. However, this case remains clinically valuable as it highlights the difficulty of recognizing neuroblastoma when it initially presents with features strongly suggestive of septic arthritis.

This case emphasizes the importance of reassessing the working diagnosis when children with presumed musculoskeletal infection fail to improve despite appropriate antimicrobial therapy. Early multidisciplinary collaboration among pediatricians, orthopedic surgeons, radiologists, and pediatric oncologists, together with timely use of whole-body MRI when clinical findings are atypical, may facilitate earlier diagnosis of metastatic neuroblastoma and reduce delays in definitive management.

## Conclusions

Neuroblastoma should remain an important differential diagnosis in children presenting with persistent fever, limping, and musculoskeletal pain, particularly when clinical improvement is not achieved despite appropriate treatment for presumed infection. Failure to respond to antimicrobial therapy, together with sterile microbiological cultures, progressive anemia, persistently elevated inflammatory markers, and diffuse marrow abnormalities on imaging, should prompt consideration of an underlying malignancy. In our patient, the combination of whole-body MRI findings and elevated urinary catecholamine metabolites strongly supported the suspicion of metastatic neuroblastoma. Although tissue confirmation could not be obtained at our institution, histopathological confirmation remains essential for definitive diagnosis and appropriate risk stratification. Maintaining a high index of suspicion and pursuing timely multidisciplinary evaluation may facilitate earlier recognition and appropriate management of children with atypical musculoskeletal presentations.
